# An exploratory study of explainable deep learning for predicting bone mineral density using clavicle features on chest radiographs: A multi‐task approach with regression and segmentation

**DOI:** 10.1002/acm2.70336

**Published:** 2025-11-23

**Authors:** Yoshiyuki Iwao, Kenshi Shiotsuki, Fumio Hashimoto, Takahiro Ochiai, Tsuneo Kagawa, Ryoichi Nagata, Michihiro Eto, Yuji Hatanaka, Yukito Yoshida, Yoshiki Asayama

**Affiliations:** ^1^ Medical Technology Department Oita University Hospital Yufu City Oita Japan; ^2^ Graduate School of Science and Engineering Chiba University Chiba Japan; ^3^ J. Crayton Pruitt Family Department of Biomedical Engineering University of Florida Gainesville USA; ^4^ Division of Computer Science and Intelligent Systems Faculty of Science and Technology Oita University Oita City Oita Japan; ^5^ Department of Health and Medical Sciences Nippon Bunri University Oita City Oita Japan; ^6^ Graduate School of Engineering Oita University Oita City Oita Japan; ^7^ Department of Radiology Oita University Faculty of Medicine Yufu City Oita Japan

**Keywords:** convolutional neural network, deep learning, explainable artificial intelligence, multi‐task learning

## Abstract

**Purpose:**

Although bone mineral density (BMD) measurement using dual‐energy x‐ray absorptiometry (DXA) is the most common method of diagnosing osteoporosis, it is not widely used to screen patients. In this exploratory study, we developed a multi‐task learning model that predicts BMD from chest radiographs using clavicular features and supports network explainability.

**Methods:**

The proposed multi‐task learning model integrates segmentation and regression tasks by incorporating a regression branch into the U‐Net architecture in an end‐to‐end manner. A total of 1600 patients who underwent chest radiography and DXA of the lumbar vertebrae were included in this study. We compared the BMD predictive performance of the mean absolute error (MAE) and Pearson correlation coefficient (*R* value) between the proposed multi‐task learning model and the single‐task learning model, which was defined as the comparison model that only performed BMD prediction. Additionally, model performance for classifying osteoporosis, osteopenia, and normal bone status was evaluated via reclassification analysis based on the World Health Organization (WHO) criteria. Confusion matrices were generated, and classwise and macro‐averaged performance metrics were calculated. To confirm the rationale for the BMD predictions, we evaluated heat maps generated using the gradient‐weighted class activation mapping technique to determine whether the highlighted regions overlapped with the clavicle.

**Results:**

The multi‐task learning model demonstrated superior predictive performance (MAE of 0.092 g/cm^2^ and *R* value of 0.769) compared with the single‐task learning model (MAE of 0.101 g/cm^2^ and *R* value of 0.724), a statistically significant (*p* < 0.001) difference in MAE. Bland–Altman analysis showed that the multi‐task learning model had good agreement with narrower limits of agreement, although a bias was present (bias: −0.013 g/cm^2^; limits of agreement: −0.248 to 0.223 g/cm^2^). In contrast, the single‐task model showed slightly wider agreement limits (bias: −0.003 g/cm^2^; limits of agreement: −0.257 to 0.252 g/cm^2^). In the reclassification analysis based on the WHO criteria, the multi‐task learning model resulted in fewer misclassifications than the single‐task learning model. The macro‐averaged sensitivity, specificity, precision, and F1 score were 0.647, 0.826, 0.680, and 0.659, respectively, for the multi‐task model, compared with 0.597, 0.809, 0.660, and 0.616, respectively, for the single‐task model. The heat maps in the multi‐task learning model highlighted different regions compared with the single‐task model, the clavicular area.

**Conclusion:**

The proposed multi‐task learning model demonstrated the predictive rationale by focusing on the clavicle in chest radiographs, which is clinically relevant to BMD, and showed improved performance compared with the single‐task model.

## 1 INTRODUCTION

Osteoporosis affects more than 200 million people worldwide and is a serious global health concern.^1^ It is a metabolic bone disease characterized by low bone mineral density (BMD) and microstructural deterioration of the bone structure, leading to a consequent increase in the risk of fragility fracture.^1^, ^2^ Fractures of the spine, hip, and wrist caused by osteoporosis often lead to disorders that reduce the quality of life of a patient. Therefore, early diagnosis is necessary to prevent such fragility fractures.^3^, ^4^ Currently, the gold standard for screening osteoporosis is BMD measurement using dual‐energy x‐ray absorptiometry (DXA).^2^ However, osteoporosis screening is inadequately widespread owing to a lack of awareness of the disease, a paucity of self‐identified symptoms, and restricted DXA accessibility.^5^ Therefore, alternative osteoporosis screening methods that use more accessible medical imaging techniques such as radiography, including spine,^6^, ^7^ hip,^8^, ^9^ dental panoramic,^10^, ^11^ and chest radiographs, have been proposed.^12^, ^13^, ^14^, ^15^, ^16^ Chest radiography is the most common diagnostic modality.^17^ Hence, chest radiography may contribute to the detection of osteoporosis.

In a previous study using chest radiographs, Kumar et al. inferred the proximal femur BMD from the clavicular cortical thickness.^12^ Furthermore, several deep learning (DL)‐based methods have been reported. For instance, Ohta et al. reported a screening method based on the classification of multiple regions of interest along the clavicle.^13^ Jang et al. performed screening based on classification issues utilizing the *T* score.^14^ Sato et al. employed ensemble learning with chest radiographs, age, and sex to predict the *T* score‐based classification and BMD.^15^ Wang et al. proposed a BMD estimation model that jointly learns multiple regions of interest, automated using bone landmarks.^16^ Some of these previous studies suggest the hypothesis that incorporating detailed anatomical features, such as the bone, can improve BMD prediction. However, they also highlight the need for models that provide clear and explainable predictions to ensure clinical trust and utility.

In the medical field, decisions emanating from DL profoundly affect patient health. Therefore, explainable artificial intelligence (XAI) is critical for enhancing reliability and safety, as well as promoting transparency in patient health‐related decisions.^18^, ^19^, ^20^ XAI supports the understanding of model decisions by visually presenting the rationale and salient features involved in the prediction process. One of the most widely used techniques is gradient‐weighted class activation mapping (Grad‐CAM),^21^ which helps visualize the regions of focus in convolutional neural networks as heat maps. This can facilitate a visual understanding of the rationale behind the predictions and salient features.^22^ In DL‐based BMD prediction, it is important that the model's focus is on the bone structures to extract clinically relevant information. If the network instead attends to the background regions outside the bone area, it suggests that the prediction may rely on non‐clinical features rather than meaningful bone attributes.

In this study, we focus on the correlation between the clavicle and BMD on chest radiographs^12^, ^13^ and explore the possibility of predicting BMD in the clavicle region. Specifically, we develop a model that utilizes multi‐task learning^23^ by incorporating clavicle segmentation as an auxiliary task within a regression‐based BMD prediction model, thereby encouraging the network to focus on clavicular features. We evaluate the predictive performance of the proposed model and assess the anatomical relevance of the regions emphasized during prediction using Grad‐CAM analysis, which was used as a visual support tool to aid the model's explainability.

## 2 MATERIALS AND METHODS

In this study, we propose a multi‐task learning model that integrates segmentation and regression by incorporating a regression branch into the U‐Net architecture.^24^ A regression branch is added at the end of the encoder. The proposed multi‐task learning model provides clinically significant BMD predictions, focusing on the clavicle, by sharing segmentation and regression features from the end of the encoder path and the regression branch.

### 2.1 Network architecture

**FIGURE 1:**
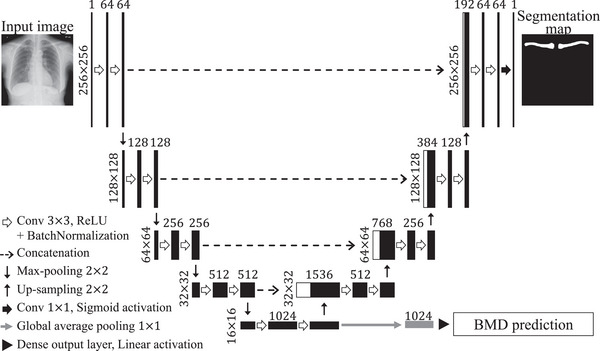
Overall architecture of the proposed multi‐task learning approach for clavicle segmentation and predictive BMD by regression.

The proposed multi‐task learning model, which integrates the segmentation and regression tasks, is illustrated in Figure 1. The network used the U‐Net architecture as the backbone, which demonstrates excellent performance in medical image segmentation.^25^

The backbone network comprises three parts: (i) an encoder path, (ii) a decoder path, and (iii) the skip connections between them. The encoder path, which is responsible for feature extraction, incorporates four down‐sampling stages. Each down‐sampling stage comprises a convolutional layer with a 3 × 3 kernel, followed by a rectified linear unit (ReLU) activation function. Batch normalization (BN) is performed after each convolutional layer. Subsequently, a 2 × 2 max‐pooling layer is introduced to reduce the spatial dimensions. The decoder path, which is responsible for up‐sampling and reconstructing the output, comprises four stages. In each up‐sampling stage, a 2 × 2 up‐sampling layer was introduced to increase the spatial dimensions. Subsequently, a 3 × 3 convolutional layer, ReLU, and BN were applied, similar to the encoder. In the final stage of the decoder path, a 1 × 1 convolution and sigmoid activation functions were applied to produce the segmented image. Skip connections were established between the corresponding layers in the encoder and decoder. These skip connections facilitate the direct flow of detailed information from lower‐resolution layers in the encoder to higher‐resolution layers in the decoder, thereby enhancing the ability of the model to accurately localize features during segmentation tasks.

A regression branch is added at the end of the encoder. The branch incorporates a global average pooling layer and a final dense output layer designed with a single unit and a linear activation function, aimed at the regression prediction of BMD. The multi‐task learning model uses a chest radiograph as the input and generates two outputs: a clavicle segmentation map and BMD prediction. The two tasks, including clavicle segmentation and BMD prediction, share features extracted from the encoder path. Therefore, the proposed multi‐task learning model was designed to predict the BMD. We selected the clavicle as the segmentation target because it is reliably depicted on standard chest radiographs, can be annotated with high reproducibility, and has been reported to be associated with BMD and osteoporosis risk in previous studies.^12^, ^13^, ^16^

### 2.2 Loss function

In the proposed multi‐task learning model, the segmentation and regression losses are linearly combined as follows:

(1) $$L_{intg}=\lambda L_{seg}+\left(1-\lambda \right)L_{reg},$$

where $L_{seg}$ denotes the segmentation loss, $L_{reg}$ denotes the regression loss, and λ denotes the loss weight of the segmentation task. In this study, the parameter λ was set to 0.8. The value of λ was determined through preliminary evaluation of segmentation attention using Grad‐CAM heat maps, as detailed in Section 2.7 (Hyperparameter settings).

For the segmentation task, segmentation loss was utilized to measure the shape similarity between the segmentation maps and the ground truth. Segmentation loss based on the Dice coefficient is defined as follows:

(2) $$L_{seg}=1-\frac{2\cdot \left|S_{pred}\cap S_{gt}\right|}{\left|S_{pred}\right|+\left|S_{gt}\right|},$$

where $S_{pred}$ and $S_{gt}$ denote the predicted segmentation map and the ground truth.

The regression loss utilized the mean squared error, which quantified the average absolute difference between the ground truth and predicted values, and serves as a measure of the predictive accuracy of the model, as follows:

(3) $$L_{reg}=\frac{1}{n}\sum {\left(BMD_{pred}-BMD_{gt}\right)}^{2},$$

where *n* denotes the total number of samples, $BMD_{gt}$ denotes the ground truth value, and $BMD_{pred}$ denotes the predicted value for the regression task.

### 2.3 Comparison model

We define a single‐task learning model as the comparison model. The architecture of the single‐task learning model comprised an encoder path and regression branches, structured similarly to the multi‐task learning model. The single‐task learning model uses a chest radiograph as the input and produces $BMD_{pred}$ as the output. In the single‐task learning model, only $L_{reg}$ is used as the loss function.

### 2.4 Grad‐CAM analysis

To assess the explainability of the proposed model, we employed Grad‐CAM for visualization. The gradient of $BMD_{pred}$ with respect to the *k*‐th feature map $A^{k}=a_{uv}^{k}\;$ in the final convolutional layer immediately preceding the regression branch is expressed as:

(4) $$\frac{\partial BMD_{pred}}{\partial A^{k}},$$

The importance weights for each feature map were then calculated as follows:

(5) $$\alpha^{k}=\frac{1}{UV}\;\sum_{u=1}^{U}\sum_{v=1}^{V}\frac{\partial BMD_{pred}}{\partial \alpha_{uv}^{k}},$$

where *U* and *V* are the spatial dimensions of the feature maps. Finally, the heat map of the Grad‐CAM is generated by using ReLU activation:

(6) $$L_{Grad}=ReLU\left(\sum_{k=1}^{K}\alpha^{k}A^{k}\right),$$

where ReLU(·) denotes the ReLU activation function.

### 2.5 Study participants

This retrospective study was approved by the institutional ethics committee, which waived the requirement for informed patient consent. This study included patients aged > 20 years who underwent posteroanterior chest radiography and DXA of the lumbar spine between January 2017 and July 2021.

Chest radiographs were obtained using a diagnostic radiography system (BENEO, Fujifilm, Tokyo, Japan). Chest radiographs were acquired using a tube voltage of 120 kVp, tube current of 160 mA, exposure time for automatic exposure control (AEC), and a source‐to‐image distance (SID) of 200 cm. These parameters reflect the standard imaging protocol used at our institution for routine chest radiography and are consistent with imaging guidelines.^26^

Bone densitometry was performed using a DXA system (QDR‐4500; Hologic, Waltham, MA, USA) with standard protocols to measure the BMDs of L2–L4 (second to fourth lumbar vertebrae). The mean value of the measured L2–L4 BMDs was used as $BMD_{gt}$ in this study.

**FIGURE 2:**
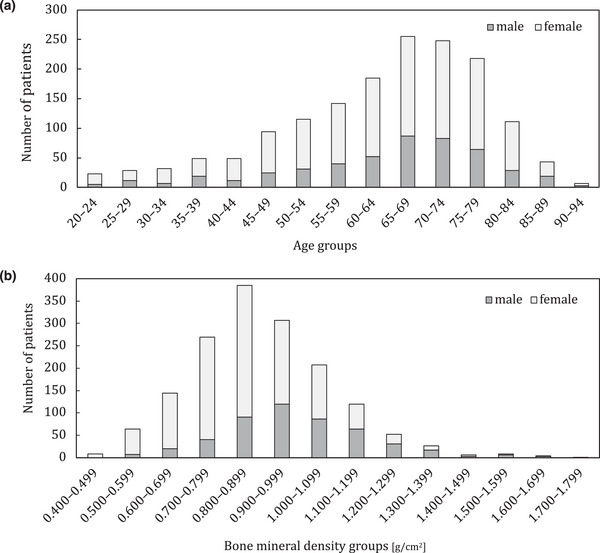
Histogram illustrating the distribution of age, sex, and bone mineral density (BMD) characteristics in the dataset: (a) age distribution by sex and (b) BMD distribution by sex.

The duration between chest radiography and DXA was 6 months, in accordance with a previous study.^15^ The dataset also included patients with implants or clinical features owing to disease within the imaging range of chest radiographs. Patients with lumbar spine conditions, such as compression fractures or scoliosis, were excluded from the study because of the possibility of inaccurate BMD measurements. The dataset included 1600 patients, within the age range of 20–91 years. The characteristics of the datasets are listed in Table 1. Figure 2 shows the detailed statistics of age, sex, and BMD in the dataset.

**TABLE 1:** Demographic characteristics of the dataset.

Participant	1600
Sex (male, female)	488 (30.5%), 1112 (69.5%)
Age (year), mean ± SD	63.3 ± 14.6
BMD (g/cm^2^), mean ± SD	0.898 ± 0.188

### 2.6 Data preparation

The original chest radiographs were 12‐bit grayscale with matrix sizes of 2373 × 2373, 2373 × 2880, 2880 × 2373, or 2880 × 2880 pixels. These images are “for‐presentation” images routinely stored in our institutional picture archiving and communication system (PACS). They were resized to a 256 × 256 matrix while preserving their aspect ratios and using zero padding. A ground truth image of clavicle segmentation was created by a radiologic technologist who manually extracted the clavicle region from chest radiographs under the guidance of a radiologist. The dataset comprised 1600 patients, randomly split into five groups of 320 cases each. 5‐fold cross‐validation was conducted by iteratively using one group as the test set, while the remaining four groups were further split into training and validation sets in a 3:1 ratio. This procedure was repeated five times, such that each group served as the test set once. Data augmentation, including random shifts, rotations, and horizontal flips, was applied to the training set.

### 2.7 Hyperparameter settings

Hyperparameters were optimized using the training and validation sets. The Adam optimizer was used with a learning rate of 0.00004 and a batch size of 16. The learning rate was determined through a grid search process in which various learning rates were tested and evaluated for their performance. The batch size was empirically determined in consideration of the computational environment. We used the same hyperparameters for both the multi‐task and single‐task learning models. These values were determined based on validation performance in the multi‐task model. The number of epochs in the multi‐task and single‐task learning models was set to 60 and 30, respectively. During training, the validation MAE was monitored at each epoch, and the model weights corresponding to the epoch with the lowest validation MAE were selected. In the multi‐task learning model, the parameter λ for loss weighting was 0.8. This value was selected through a preliminary examination in which λ was systematically varied from 0.5 to 0.9. The degree of attention to the clavicle was evaluated using the Grad‐CAM heat maps, selecting the λ that resulted in the highest proportion of cases with heat maps overlapping the clavicle region. This preliminary analysis was conducted using only the training and validation datasets, thereby avoiding contamination of the test results.

### 2.8 Evaluation

In both the multi‐task and single‐task learning models, the mean absolute error (MAE) and Pearson correlation coefficient (*R* value) between $BMD_{pred}$ and $BMD_{gt}$ were used as measures of performance in BMD prediction. MAE represents the average of the absolute differences between $BMD_{pred}$ and $BMD_{gt}$ providing a measure of prediction accuracy, and is calculated using the following formula:

(7) $$MAE=\frac{1}{n}\;\sum \left|BMD_{pred}-BMD_{gt}\right|,$$

The *R* value indicates the strength of the linear correlation between $BMD_{pred}$ and $BMD_{gt}$. The MAEs and *R* values were calculated using all the test data. After calculating these metrics for both the multi‐task and single‐task learning models, we compared the results to determine the method that provided the most accurate and correlated predictions. Furthermore, we calculated the Dice coefficient between the ground truth segmentation labels and the predicted segmentation maps output from the segmentation task. In addition, Bland–Altman analysis was performed to evaluate the degree of agreement between $BMD_{pred}$ and $BMD_{gt}$. The Bland–Altman analysis involves calculating the bias and limits of agreement. Bias represents the average difference between the two models, indicating systematic discrepancies. The limits of agreement provide insight into the variability and potential discrepancies in the predictions by illustrating the expected range of differences for 95% of the data points. If the limits of agreement are narrow, the two methods are in good agreement, with a small variation between their predictions. To assess local trends in prediction error across the spectrum of BMD values, we applied locally weighted scatterplot smoothing (LOWESS) to the absolute error values. LOWESS is a non‐parametric technique that fits locally weighted linear regressions to subsets of the data, enabling flexible visualization of non‐linear relationships without assuming a global functional form. In this study, LOWESS was used as a post hoc analysis tool to explore how the absolute prediction error varied as a function of $BMD_{gt}$, independent of model architecture. Furthermore, both $BMD_{gt}$ and $BMD_{pred}$ were converted to *T* scores, which were calculated as follows:

(8) $$T\;score=\frac{BMD-YAM}{SD}\;,$$

where YAM denotes the sex‐specific young adult mean of BMD and SD denotes the corresponding standard deviation. In our clinical environment, the YAM and SD values adopted were 1.024 and 0.131 g/cm^2^ for males, and 1.010 and 0.119 g/cm^2^ for females.^27^ Based on the World Health Organization (WHO) criteria,^28^ *T* scores were classified into three diagnostic classes: normal (*T* score ≥ −1.0), osteopenia (−2.5 < *T* score < −1.0), and osteoporosis (*T* score ≤ −2.5). In addition, model performance for this three‐class classification task was evaluated using a confusion matrix. For each class, the sensitivity, specificity, precision, and F1 score were calculated, and macro‐averaged values were also reported.

To evaluate the anatomical relevance of the model's attention, we performed a quantitative analysis using heat maps of the Grad‐CAM. The regions with heat map values exceeding 50% of the maximum values were highlighted. An evaluation was thereafter performed to determine whether an overlap was present between the highlighted areas in the heat map and clavicle segmentation map.

The neural network implementation, evaluation, and statistical analysis were performed on a computer with Windows 10 Pro as the operating system, a central processing unit (Intel Core i9‐10900 KF at 3.0 GHz), and a graphics processing unit (NVIDIA GeForce RTX 3080 with 12 GB of memory). The software environment comprised Python 3.8.5 as the programming language, using Keras 2.5.0 with a TensorFlow 2.5.0 backend as the DL framework.

### 2.9 Statistical analysis

To assess the differences in predictive performance between the multi‐task and single‐task learning models, we compared the MAEs using the Wilcoxon signed‐rank test, as the error distributions were not normally distributed (as verified by the Shapiro–Wilk test). A *p*‐value of less than 0.05 was considered statistically significant. All statistical analyses were conducted using the SciPy and Pingouin libraries in Python.

## 3 RESULTS

**FIGURE 3:**
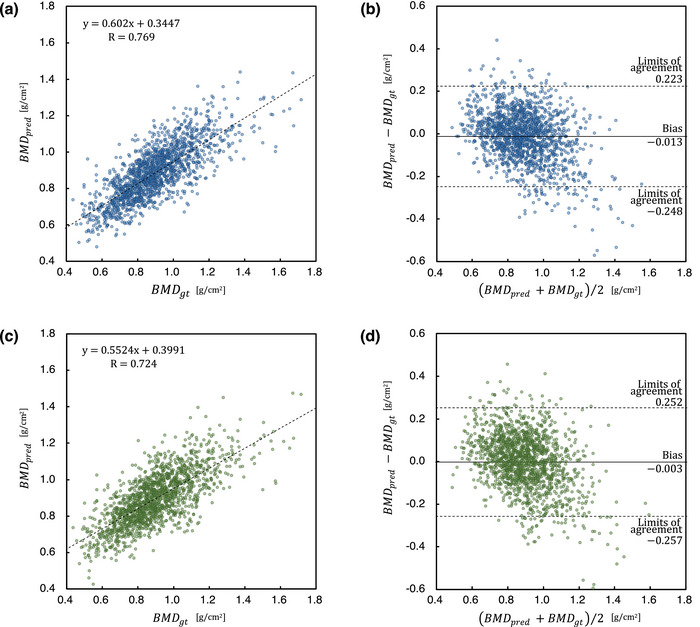
Scatter plots and Bland–Altman plots for comparison of prediction values (*BMD_pred_*) with the ground truth values (BMD_*gt*_) on the integrated data during cross‐validation for each model; multi‐task learning model (**a**, **b**) and single‐task learning model (**c, d**).

**FIGURE 4:**
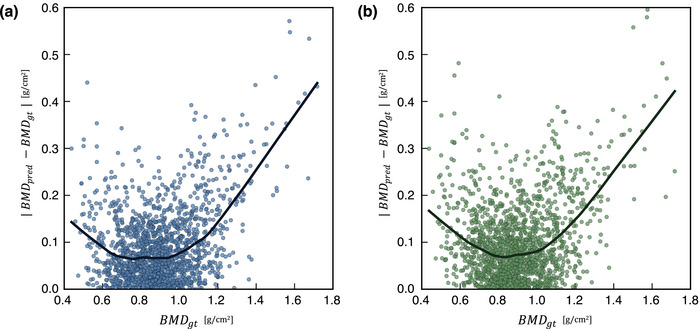
Absolute errors between the *BMD_pred_* and *BMD_gt_* (vertical axis) plotted against the *BMD_gt_* (horizontal axis), for (a) the multi‐task and (b) single‐task learning models, respectively. Each dot represents an individual subject. The solid line in each subfigure represents a locally weighted scatterplot smoothing (LOWESS) curve.

**FIGURE 5:**
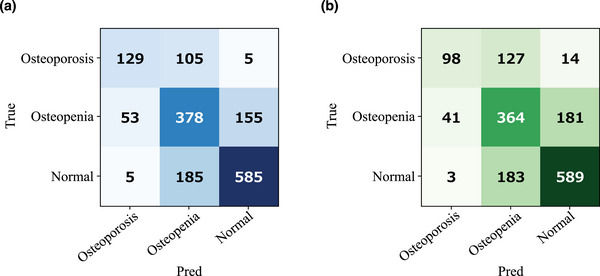
Confusion matrices for osteoporosis classification using the multi‐task learning model (a) and the single‐task learning model (b). The rows represent the ground truth classes and the columns represent the predicted classes, based on WHO criteria: Osteoporosis (*T* score ≤ −2.5), Osteopenia (−2.5 < *T* score < −1.0), and Normal (*T* score ≥ −1.0).

The MAEs and *R* values for the multi‐task and single‐task learning models are listed in Table 2. The multi‐task learning model performed better than the single‐task learning model for all folds during 5‐fold cross‐validation. Overall, the multi‐task learning model achieved a significantly lower MAE (0.092 vs. 0.101 g/cm^2^; *p* < 0.001; Wilcoxon signed‐rank test) compared to the single‐task model. The corresponding *R* values were 0.769 for the multi‐task model and 0.724 for the single‐task model. For the multi‐task learning model, the Dice coefficient between the ground truth segmentation labels and the predicted segmentation maps from the segmentation task was 0.960 ± 0.028. Scatter plots showing the correlation between $BMD_{gt}$ and $BMD_{pred}$ for both multi‐task and single‐task learning models, along with Bland–Altman plots, are shown in Figure 3. The scatter plots exhibited a positive correlation between $BMD_{gt}$ and $BMD_{pred}$. The Bland–Altman analysis exhibited a bias of –0.013 for the multi‐task learning model, with 95% limits of agreement ranging from –0.248 to 0.223. For the single‐task learning model, a bias of –0.003 was obtained, with 95% limits of agreement ranging from –0.257 to 0.252. Figure 4 shows the absolute prediction errors plotted against $BMD_{gt}$ for the multi‐task and single‐task learning models. In both models, the LOWESS curves indicate an increasing trend in absolute error with higher $BMD_{gt}$. The confusion matrices based on reclassification according to the WHO criteria for the multi‐task and single‐task learning models are shown in Figure 5. Table 3 summarizes the classwise and macro‐averaged sensitivity, specificity, precision, and F1 score.

**TABLE 2:** Mean absolute error and Pearson correlation coefficient of each model as the BMD prediction performance.

		Fold_1	Fold_2	Fold_3	Fold_4	Fold_5	Overall
MAE [g/cm^2^]	Multi‐task learning model	0.084	0.095	0.097	0.091	0.091	0.092
	Single‐task learning model	0.089	0.103	0.109	0.097	0.103	0.101
*R*	Multi‐task learning model	0.791	0.766	0.764	0.789	0.746	0.769
	Single‐task learning model	0.744	0.732	0.729	0.755	0.680	0.724

MAE; mean absolute error, *R*; Pearson correlation coefficient (*R*‐value).

**TABLE 3:** Classwise and macro‐averaged evaluation metrics between multi‐task and single‐task learning models for osteoporosis classification.

		Sensitivity	Specificity	Precision	F1 score
Osteoporosis	Multi‐task learning model	0.540	0.957	0.690	0.606
	Single‐task learning model	0.410	0.968	0.690	0.514
Osteopenia	Multi‐task learning model	0.645	0.714	0.566	0.603
	Single‐task learning model	0.621	0.694	0.540	0.578
Normal	Multi‐task learning model	0.755	0.806	0.785	0.770
	Single‐task learning model	0.760	0.764	0.751	0.756
Average	Multi‐task learning model	0.647	0.826	0.680	0.659
	Single‐task learning model	0.597	0.809	0.660	0.616

**FIGURE 6:**
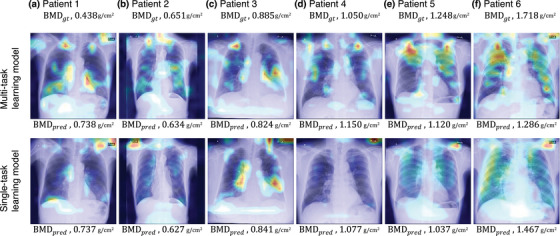
Example of a heat map based on Grad‐CAM comparison between a multi‐task learning model (top row) and a single‐task learning model (bottom row) for patients with various BMD values. Each column corresponds to a different patient within the dataset, with patient 1 representing the minimum BMD (a), patient 3 representing the median BMD (c), and patient 6 representing the maximum BMD (f). Patients 2, 4, and 5 are randomly selected patients with intermediate BMD values (b,d,e). The red areas on the heat map indicate the regions most focused on by the network model. *BMD_gt_* and *BMD_pred_* indicate the BMD of the ground‐truth value and the prediction value.

Representative heat maps for the multi‐task and single‐task learning models are shown in Figure 6. These heat maps overlay a bright red color on chest x‐rays, visualizing the area most relevant to BMD prediction. In the multi‐task learning model (Figure 6, top row), the clavicles were prominently emphasized. In contrast, highlighted areas were observed around the background and the radiopaque marker periphery in the single‐task learning model (Figure 6, bottom row). The areas highlighted in the heat maps differed between the multi‐task and single‐task learning models, even for the same patient. The number of cases in which an overlap was present between the heat map and clavicle segmentation map was 86.9% (1391/1600 cases) for the multi‐task learning model, compared to 66.6% (1065/1600 cases) for the single‐task learning model.

## 4 DISCUSSION

The proposed multi‐task learning model directs the network's attention in BMD prediction, thereby focusing on the clavicular region of chest radiographs and supporting explainability by providing a rationale for its predictions. The heat maps illustrating the rationale for the predictions indicate distinct highlighted areas for the multi‐task and single‐task learning models. The quantitative evaluation of the overlap between the heat maps and clavicle segmentation maps showed that, as demonstrated in the example in Figure 6, the multi‐task learning model had more instances of highlighting the clavicle. This is because the multi‐task learning model shares features between the tasks of clavicle segmentation and BMD prediction through regression in an end‐to‐end manner, with the segmentation task functioning as an attention mechanism that tunes the feature extractor to focus on the image features in the clavicle region. Grad‐CAM highlights the clavicle in the multi‐task learning model, demonstrating that the rationale for BMD prediction is rooted in the clavicle. Previous studies have demonstrated that bone regions on chest radiographs, including the clavicle, are critical for accurate BMD assessment.^13^, ^16^ The present study aligns with these clinical expectations. Therefore, the multi‐task learning approach may support the explainability of BMD predictions by providing a rationale focused on the clavicle. The BMD prediction by the multi‐task learning model outperformed that by the single‐task learning model in terms of both the MAE and *R* value. The results were attributed to the properties of multi‐task learning, which combines multiple pieces of information from different tasks to improve performance and generalization.^23^ Furthermore, the Bland–Altman analysis evaluated the agreement between the predicted and measured values for both models. In this analysis, the multi‐task model demonstrated narrower limits of agreement (−0.248 to 0.223 g/cm^2^) than the single‐task model (−0.257 to 0.252 g/cm^2^), indicating more consistent predictions. Although the multi‐task model showed a slightly larger bias than the single‐task model (−0.013 vs. −0.003 g/cm^2^), the absolute bias values for both models were small and not considered clinically significant. Notably, the single‐task model corresponds to a multi‐task framework in which the segmentation loss weight (λ) was effectively set to zero. Therefore, the observed increase in bias can be attributed to incorporating the segmentation task. However, this study did not directly evaluate the impact of segmentation‐related errors on BMD prediction. Further investigation is required to clarify this relationship in future work. By combining confusion matrices with performance metrics such as sensitivity, specificity, precision, and F1 score, we conducted a detailed evaluation of model performance. The multi‐task learning model outperformed the single‐task learning model and demonstrated a clinically important advantage in reducing missed diagnoses. In particular, from a clinical perspective, the direction of misclassification is critical. Reclassifying normal cases as osteoporosis may lead to additional diagnostic workup or unnecessary treatment, but it is generally less harmful than the opposite scenario. By contrast, misclassifying true osteoporosis or osteopenia as normal can delay diagnosis and result in missed opportunities for fracture prevention. In this study, compared with the single‐task learning model, the multi‐task learning model resulted in lower rates of underclassification of osteoporosis as normal (2.1% vs. 5.9%) and osteopenia as normal (26.5% vs. 30.9%), suggesting its potential to minimize clinically significant missed diagnoses. However, in both models, a considerable proportion of osteoporosis cases were downgraded to osteopenia, indicating the need for further refinement to reduce underestimation in high‐risk patients.

As shown in Figure 3, a tendency exists to underestimate the number of patients with higher BMD. This is due to the lack of data for patients with high BMD, as shown in Figure 2, which results in an imbalanced dataset. Whereas the performance of this model is adequate for the early detection of patients with low BMD, such as those with potential osteopenia or osteoporosis, addressing the dataset imbalance is crucial for further improving the accuracy and reliability. In future studies, we intend to address this imbalance to enhance the performance of the model. Compared with the results of Wang et al.^16^ and Sato et al.,^15^ who predicted lumbar BMD from chest radiographs using DL, the results of this study are moderate (previous studies: *R* = 0.884, 0.63; this study: *R* = 0.771). Wang et al. achieved high prediction performance for BMD using multiple bone regions as input images. This finding suggests that BMD‐related features can be obtained from bones other than the clavicle. We could also apply multi‐task learning by incorporating bones other than the clavicle into our segmentation task.

The advantage of this model is that the segmentation maps are used only during training and are not necessary for clinical use. This simplifies the workflow by eliminating the need for additional preprocessing steps, such as clavicle region extraction, thereby enhancing practicality and efficiency. However, challenges still exist, such as issues with device connectivity and ethical concerns, in directly inputting images from the imaging system or PACS into the DL model immediately after radiography. To achieve a more seamless clinical application, addressing the interoperability issues between the imaging system or PACS and the DL model will be necessary.

Furthermore, when expressed in terms of the *T* score used in clinical practice, the MAE observed in our study (0.092 g/cm^2^) corresponds to a *T* score variation of approximately 0.7–0.8, depending on sex. This magnitude of error may need to be considered when calculating the *T* score. To further investigate this issue, we conducted a supplementary LOWESS analysis to examine how prediction errors vary across the $BMD_{gt}$ range. As shown in Figure 4, absolute prediction errors tend to increase with higher $BMD_{gt}$, contributing significantly to the overall MAE. In contrast, the absolute errors are relatively small within the $BMD_{gt}$ range of approximately 0.6–1.0 g/cm^2^. Notably, this range includes key diagnostic thresholds. For example, BMD ≈ 0.713 g/cm^2^ (corresponding to *T* score = –2.5) marks the boundary between osteoporosis and osteopenia, whereas BMD ≈ 0.891 g/cm^2^ (corresponding to *T* score = –1.0) represents the boundary between osteopenia and normal bone mass. This trend suggests that prediction errors may have a relatively limited impact on diagnostic classification near these clinically important thresholds. This study primarily aimed to improve the explainability and predictive accuracy of BMD prediction by employing a multi‐task learning framework that encourages the model to focus on anatomically meaningful regions, such as the clavicle. By doing so, the proposed method enhances the transparency and reliability of the prediction process.

This study has several limitations. The retrospective design used in this study incorporated only imaging data from a single medical institution. External validation using datasets from other medical institutions has not yet been performed. Jang et al.^14^ reported no significant reduction in screening accuracy after external validation. In standardized patient positioning and acquisition protocols, chest radiographs consistently project the lungs, heart, and bones, and thus present similar anatomical image layouts across institutions.^29^ This consistency enables the proposed model for clavicle segmentation to be applicable to a wide range of chest radiographs. However, we acknowledge that other imaging factors, such as grid usage, grid frequency, SID, AEC settings, detector type, and post‐processing algorithms, may vary considerably between institutions and equipment types. These variations can affect the image quality and pixel intensity distributions, which may, in turn, influence the performance and generalizability of the DL models. In particular, the current study utilized “for‐presentation” images that had undergone vendor‐specific post‐processing for clinical display. Although these images reflect real‐world clinical use, the presence of post‐processing may limit their generalizability across vendors and systems. The use of “for‐processing” images with more standardized and unprocessed pixel values may improve model consistency and should be considered in future studies. Additionally, the learning rate was determined via a grid search on the training and validation sets using the multi‐task model, and the same value was used for both multi‐task and single‐task models to ensure a fair comparison. This consistent approach across models contributed to robust performance in our experiments. To optimize the balance between the segmentation and regression tasks, λ was systematically evaluated by varying its value from 0.5 to 0.9 on the training and validation datasets. An optimal value of 0.8 was selected based on the degree of overlap between the Grad‐CAM maps and the ground truth clavicular regions. Future work will explore the interactions among hyperparameters to further refine model performance. Furthermore, this study focused on the clavicle as the auxiliary segmentation target; however, osteoporosis is systemic, and other skeletal regions may also carry predictive signals. Our findings are limited to lumbar spine BMD because chest radiographs were paired only with lumbar DXA. Future work should extend the segmentation beyond the clavicle and evaluate associations with the proximal femur or overall BMD. Finally, several studies have shown that Grad‐CAM may not consistently highlight diagnostically relevant regions and can sometimes yield misleading interpretations.^30^–^32^ Therefore, the use of Grad‐CAM in medical image interpretation should be recognized as having important limitations. In this exploratory study, Grad‐CAM is not a definitive XAI method but one of many explainability tools, and its outputs should be interpreted with caution as preliminary evidence rather than definitive proof. Future work should include more rigorous, quantitative evaluations to strengthen its role in XAI.

## 5 CONCLUSION

In this study, we proposed a novel multi‐task learning model that supports explainable BMD prediction using clavicle features on chest radiographs. The heat map visualizations revealed that the multi‐task learning model predicts BMD by focusing on the clavicular region. Although Grad‐CAM‐based explainability should be interpreted as preliminary evidence, this bone‐focused attention enables the model to generate clinically meaningful BMD predictions. Furthermore, the BMD prediction performance was improved by increasing the generalization capability through multi‐task learning. This study provides new potential for osteoporosis screening using DL with chest radiography.

## AUTHOR CONTRIBUTIONS

*Study design*: Yoshiyuki Iwao, Kenshi Shiotsuki, and Fumio Hashimoto. *Data collection and data preprocessing*: Takahiro Ochiai, Yoshiyuki Iwao, and Kenshi Shiotsuki. *Supervised data collection, annotation, and labeling*: Yukito Yoshida and Yoshiki Asayama. *Deep learning model development*: Yoshiyuki Iwao, Kenshi Shiotsuki, Fumio Hashimoto, Tsuneo Kagawa, Ryoichi Nagata, and Michihiro Eto. *Manuscript writing and editing*: Yoshiyuki Iwao, Kenshi Shiotsuki, Fumio Hashimoto. *Manuscript review and editing*: Tsuneo Kagawa, Ryoichi Nagata, Michihiro Eto, Yuji Hatanaka, Yukito Yoshida, and Yoshiki Asayama. *Supervised the development of the deep learning model*: Yuji Hatanaka

## CONFLICT OF INTEREST STATEMENT

The authors declare no conflicts of interest.

## ETHICS STATEMENT

This study was approved by the Ethics Committee of the Oita University Faculty of Medicine (Approval Number: 1946). The need for informed consent was waived due to the retrospective nature of the study.

## Data Availability

The code used in this study is available from the corresponding author upon reasonable request. However, patient data from this study cannot be shared publicly due to privacy and ethical restrictions.
